# The Evidence Base For Psychiatric Support For Living Independently And Being Included In The Community

**DOI:** 10.1192/j.eurpsy.2022.61

**Published:** 2022-09-01

**Authors:** A. Mucci

**Affiliations:** Univeristy of Campania Luigi Vanvitelli, Department Of Psychiatry, Naples, Italy

## Abstract

Functional recovery of subjects with schizophrenia remains an unmet need despite the availability of effective pharmacological and psychosocial treatments. The focus of recovery-oriented approaches is on fostering hope and resilience, fighting self-stigma, supporting self-determination and promoting social inclusion. The implementation of recovery-oriented plans requires an in depth understanding of key factors influencing real-life functioning, health status and quality of life. Recently published data from the Italian Network for Research on Psychoses have provided evidence that baseline variables associated with functional outcome at follow-up included domains not routinely assessed and targeted by intervention programs in community mental health services. As pointed out by experts in schizophrenia research and care, the management of subjects with schizophrenia has not significantly improved and only a minority of them receives integrated and personalized treatments. Shared decision-making and integrated pharmacological and psychosocial treatments, tailored on subjects’ needs, might significantly improve the outcome of subjects with schizophrenia, supporting independent living and inclusion in the community.

**Disclosure:**

Honoraria, advisory board, or consulting fees from Angelini, Astra Zeneca, Bristol-Myers Squibb, Gedeon Richter Bulgaria, Innova-Pharma, Janssen Pharmaceuticals, Lundbeck, Otsuka, Pfizer, and Pierre Fabre, for services not related to this abstract

